# Supplementary material for: The structural connectome constrains fast brain dynamics
Source: eLife. 2021 Jul 9;10:e67400. doi: 10.7554/eLife.67400 (PMC8294846; doi:10.7554/eLife.67400)
Supplement: Supplementary file 1. [file elife-67400-supp1.docx]

**Supplementary File .1 Correlations between the structural connectome and frequency-specific transition matrices.**

|  | **R** | **p** |
| --- | --- | --- |
| **Delta (0.5 – 4 Hz)** | **0.38** | **2.021e-120** |
| **Theta (4 – 8 Hz)** | **0.35** | **5.34e-100** |
| **Alpha (8 – 13 Hz)** | **0.38** | **1.46e-116** |
| **Beta (13 – 30 Hz)** | **0.38** | **7.30e-122** |
| **Gamma (30 – 48 Hz)** | **0.39** | **1.32e-123** |
